# Dietary Organic Acids Modulate Gut Microbiota and Improve Growth Performance of Nursery Pigs

**DOI:** 10.3390/microorganisms9010110

**Published:** 2021-01-05

**Authors:** Xiaoyuan Wei, Kristopher A. Bottoms, Hans H. Stein, Laia Blavi, Casey L. Bradley, Jon Bergstrom, Joshua Knapp, Robert Story, Charles Maxwell, Tsungcheng Tsai, Jiangchao Zhao

**Affiliations:** 1 Department of Animal Science, Division of Agriculture, University of Arkansas, Fayetteville, AR 72701, USA; xw010@uark.edu (X.W.); kbottoms@uark.edu (K.A.B.); jpknapp67@gmail.com (J.K.); rstory@uark.edu (R.S.); cmaxwell@uark.edu (C.M.); ttsai@uark.edu (T.T.); 2 Department of Animal Science, University of Illinois Urbana-Champaign, Champaign, IL 61801, USA; hstein@illinois.edu (H.H.S.); laiablavi@gmail.com (L.B.); 3 The Sunswine Group, LLC, Lowell, AR 72745, USA; casey@vitechusa.com; 4 Animal Nutrition and Health, DSM Nutritional Products, Parsippany, NJ 07054, USA; jon.bergstrom@dsm.com

**Keywords:** benzoic acid, gut microbiota, growth performance, weaning pigs, sodium butyrate

## Abstract

Feed additives have been suggested to improve animal growth performance through modulating the gut microbiota. The hypothesis of this study was that the combination of two organic acids would exert synergistic effects on the growth performance and gut microbiota of weaning pigs. To test this hypothesis, we followed 398 weaning pigs from two university experiment stations (University of Illinois at Urbana-Champaign (UIUC) and University of Arkansas (UA)) to determine the effects of increasing levels (0%, 0.035%, 0.070%, and 0.105%) of sodium butyrate combined with 0.5% benzoic acid on the growth performance of nursery pigs. At the UA, an additional negative control diet was included and the gut microbiota analysis was carried out. At both universities, increasing levels of sodium butyrate in a diet containing 0.5% benzoic acid improved growth performance, which reached a plateau in the pigs fed 0.035% (SBA0.035) or 0.070% (SBA0.070) butyrate. Gut microbiota analysis revealed that pigs fed the SBA0.035 diet had more diverse microbiota and contained more potentially beneficial bacteria such as *Oscillospira*, *Blautia*, and *Turicibacter* and reduced levels of *Veillonella* and *Sarcina*. Results of the present study indicated that the inclusion of sodium butyrate at moderate levels in a diet containing 0.5% benzoic acid improved growth performance of weaning pigs and established potential health benefits on gut microbiota.

## 1. Introduction

Acidifying stomach contents can delay gastric emptying and stimulate pancreatic enzyme secretion, allowing further digestion and absorption of protein and other nutrients [1]. Organic acids have been used to improve weanling pig health and growth performance due to their ability to reduce gastrointestinal tract pH, improve nutrient digestibility, and inhibit pathogenic bacterial proliferation [2]. Results of numerous experiments have documented that lowering gastrointestinal tract pH with acidifiers may stimulate pepsin activity in the stomach, which improves protein and amino acid digestion [3]. Positive responses in animal growth performance have been observed when organic acids were supplemented in diets or drinking water [4,5,6,7]. In addition to aiding nutrient digestion, growth-promoting effects observed from adding organic acids to pig diets may be due to their ability to reduce the presence of pathogenic bacteria [8,9]. This is a result of enhanced macrophage antimicrobial activity [10], which may reduce the coliform burden throughout the gastrointestinal tract [5,11,12]. Moreover, undissociated organic acid molecules can diffuse across the bacterial cell membrane and release protons (H^+^). Bacteria need to consume energy to eliminate the excess protons. The remaining anion RCOO^−^ disrupts DNA and RNA synthesis. The combination of these two actions inhibits bacterial replication and growth, leading to bacteriostatic or bactericidal effects [13]. Functions and properties vary between different types of acidifiers. For instance, butyrate is shown to modulate the immune system and to provide an instant energy source for the animal [14,15,16], while benzoic acid is more promising regarding its ability to increase nutrient digestibility, inhibit pathogenic bacteria, and maintain homeostasis of gut microbiota [17,18,19].

Previous experiments provided information on only a limited set of microbial taxa, and a gap in the understanding of how organic acids alter the entire gut microbiome exists. Therefore, two experiments were conducted to test the hypothesis that an organic dietary mixture (benzoic acid + sodium butyrate) will not only improve growth performance of weaning pigs, but also modulate the intestinal microbiota.

## 2. Materials and Methods

### 2.1. Animal Management and Experimental Design

**Figure 1:**
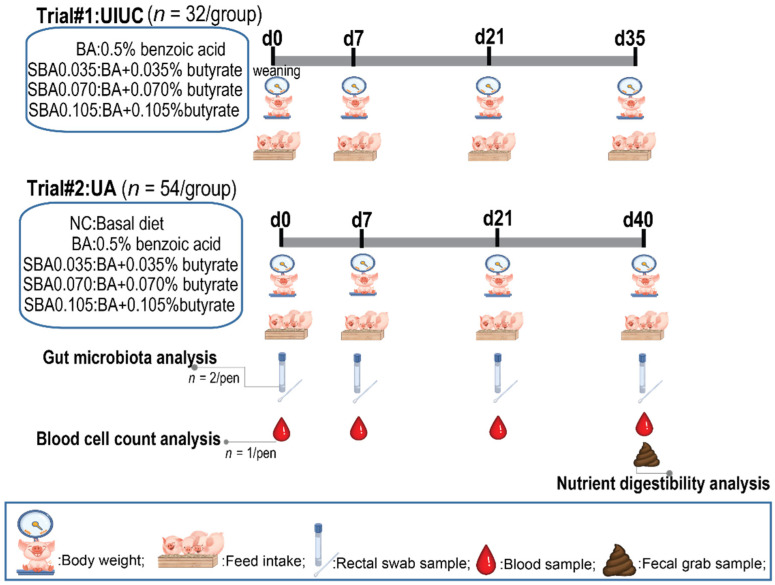
Experimental design. Trial #1 and trial #2 were conducted at the University of Illinois at Urbana-Champaign (UIUC) and the University of Arkansas (UA), respectively.

Animal management and care were approved by the Institutional Animal Care and Use Committee of the University of Arkansas (IACUC #18132) and at the University of Illinois at Urbana-Champaign (IACUC #16093). The experimental design is illustrated in Figure 1.

#### 2.1.1. Trial #1: University of Illinois at Urbana-Champaign (UIUC)

A total of 128 pigs (21 ± 1 d of age; body weight (BW): 6.89 ± 0.76 kg) were transferred to a total confinement facility on the day of weaning. Pigs were allotted to four dietary treatments with eight replicate pens per treatment. Each pen housed four pigs, two gilts and two barrows. Pens were fully slatted (each 1.2 × 1.4 m^2^), and each pen was equipped with a feeder and a nipple drinker. Room temperature was controlled. Ambient temperature was set at 30 °C upon pig arrival and was reduced by two degrees per week until a 24 °C setting for the housing temperature was achieved by the end of the study. Fluorescent lighting was provided 24 h per day during the entire study.

#### 2.1.2. Trial #2: University of Arkansas (UA)

A total of 270 pigs were transferred at weaning (21 ± 1 d of age; BW: 4.70 ± 0.60 kg) to a total confinement facility. Pigs were stratified by initial body weight and allotted to five dietary treatments. Each treatment group consisted of nine replicate pens with six pigs per pen. An attempt was made to have equal gender distribution within each pen. Pigs were housed in 1.50 × 1.20 m^2^ plastic floor pens with ad libitum access to feed and water for the duration of the experiment. Ambient temperature was set at 30 °C upon pig arrival and was reduced by two degrees per week until a 24 °C setting for the housing temperature was achieved by the end of the study. Fluorescent lighting was provided 24 h per day during the entire study.

### 2.2. Diets and Feeding

Sow milk was the sole source of nutrients for piglets prior to weaning. Upon weaning, a three-phase feeding program was used for both universities (Table 1). The experimental design is illustrated in Figure 1. Sodium butyrate (Villimax^®^, DSM Nutritional Products Inc, Parsippany, NJ, USA) and benzoic acid (Vevovitall^®^, DSM Nutritional Products Inc, Parsippany, NJ, USA) were used in the experiment. In each phase, there were four dietary treatments: BA (basal diet + 0.5% benzoic acid), SBA0.035 (BA + 0.035% sodium butyrate), SBA0.070 (BA + 0.070% sodium butyrate), and SBA0.105 (BA + 0.105% sodium butyrate). An additional (fifth) dietary treatment that was devoid of any added organic acids (NC) was used in the trial at the UA to determine the impact of 0.5% benzoic acid.

All diets were formulated to meet or exceed nutrient requirements (NRC, 2012) for pigs [20]. Pigs were fed experimental diets for 35 d and 40 d at UIUC and UA, respectively. Phase 1 diets were fed for 7 d (d 0–7), phase 2 diets were fed for 14 d (d 7–21), and phase 3 diets were fed for 14 d (d 21–35) or 19 d (d 21–40). Diets were manufactured at each institute. Titanium dioxide (0.3%; TiO2) was added to the phase 3 diet as an indigestible marker to determine the nutrient digestibility in trial #2.

### 2.3. Data Recording and Sample Collection

Pig weights were recorded at the start of the experiment and on the last day of each phase. The amount of feed offered to each pen was recorded daily and the amount of feed left in the feeder was recorded on the last day of each phase. Data were summarized to calculate average daily gain (ADG), average daily feed intake (ADFI), and average gain/feed (G:F) for each dietary treatment.

In addition to growth performance data, pen fecal grab samples, blood, and fecal swabs were collected during trial #2 at the University of Arkansas to evaluate effects of dietary treatments on nutrient digestibility, complete blood cell count (CBC), and gut microbiome. Fresh fecal grab samples were collected from each pen for two consecutive days at the end of the study (d 40) and were stored at −20 °C until analyzed. Blood samples (n = 45) for each phase were collected via jugular vein puncture into a 10 mL K2-EDTA vacutainer tube for the leukocyte differential analysis using a Hemavet 950 (Drew Scientific, Miami Lakes, FL, USA) at the beginning of the experiment and at the end of each phase to determine the complete blood cell count (d 0, 7, 21, and 40). The piglet in each pen with a BW closest to the pen-average was used, and an attempt was made to select the same gender within blocks.

Fecal swab (Puritan Opti-Swab, Puritan Medical Products, Guilford, ME, USA) samples (n = 18/treatment) were individually collected from the rectum of each animal (two median BW pigs from each pen) on days 0, 7, 21, and 40 and stored at −80 °C before DNA extraction.

### 2.4. Chemical and Statistical Analysis

#### 2.4.1. Trial #1: University of Illinois

##### Chemical Analysis

All diets were analyzed for dry matter (method 930.15; AOAC Int., (Rockville, MD, USA) 2007) and for ash (Method 942.05: AOAC Int., 2007). Diets were also analyzed for gross energy on an isoperibol bomb calorimeter (Model 6400, Parr Instruments, Moline, IL) using benzoic acid as the internal standard. The concentration of nitrogen in all diets was measured via the combustion procedure (method 999.03: AOAC Int., 2007) using a LECO FP628 analyzer (LECO Corp., Saint Joseph, MI, USA). Aspartic acid was the calibration standard and CP was calculated as N × 6.25 (Table 1).

##### Statistical Analysis

Normality of data was verified and outliers were identified using the UNIVARIATE procedure (version 9.3, SAS Institute; Cary, NC, USA). Outliers were defined as the values that deviated from the treatment mean by more than three times the interquartile range. Data were analyzed by ANOVA using the PROC MIXED of SAS in a completely randomized design with a pen as the experimental unit. The statistical model included the fixed effect of dietary treatment. Least square means were calculated for each independent variable and means were separated using the PDIFF procedure in SAS. Levels of sodium butyrate were used in the interactive matrix language procedure of SAS to generate coefficients for orthogonal contrast for treatment BA (basal diet + 0.5% benzoic acid), SBA0.035 (BA + 0.035% sodium butyrate), SBA0.070 (BA + 0.070% sodium butyrate), and SBA0.105 (BA + 0.105% sodium butyrate). Orthogonal contrasts were also used to determine linear and quadratic effects of butyrate level on growth performance. Statistical significance and tendencies were considered at *p* < 0.05 and 0.05 ≤ *p* < 0.10, respectively.

#### 2.4.2. Trial #2: University of Arkansas

##### Chemical Analysis

Fecal samples were analyzed for fecal volatile fatty acid (VFA) content via gas chromatography (Hewlett Packard 5890 Series II Gas Chromatograph, Wilmington, DE) by using 1 g of fresh fecal samples. Diets and fecal samples were dried in a drying oven (Shel Lab, model SMO28-2, Cornelius, OR, USA) at 55 °C and were then ground through a 2 mm screen in a Wiley Mill Grinder (Arthur H. Thomas, Philadelphia, PA, USA). Ground samples were then dried in an oven (BWR Scientific Gravity Oven, model 1370 GM, Radnor, PA) at 103 °C overnight to determine dry matter content using AOAC Official Method 930.15 (AOC International, Rockville, MD, USA). Dried, ground fecal, and feed samples were ashed in a furnace (Thermolyne/Sybron Ashing Oven, model FA1938) at 600 °C for 8 h and were analyzed for ash content (Ash) using AOAC Official Method 942.05 (AOC International, Rockville, MD, USA). Neutral detergent fiber (NDF) and acid detergent fiber (ADF) were analyzed according to the batch procedures outlined by ANKOM Technology Method 13 (ANKOM Technology, Macedon, NY, USA) and ANKOM Technology Method 12 (ANKOM Technology, Macedon, NY, USA), respectively, using an ANKOM 200/220 Fiber Analyzer (ANKOM Technology, Macedon, NY, USA). Nitrogen was determined via the Dumas combustion method and was analyzed with a CHN analyzer (Na-2000 N-Protein, Fisons Instruments S.p.A., Rodano (MI), Italy). Gross energy (GE) was analyzed via rapid combustion procedure using a calorimeter (Parr 6200 Calorimeter, Moline, IL, USA). Calcium and phosphorus were analyzed using the methods established by Jones et al., 1990 [21]. Acid digestion was conducted on an Environmental Express Hot Block (Charleston, SC, USA) and the resulting digesta were analyzed on an inductively coupled plasma atomic emission spectrophotometer (Spectro Arcos 160 SOP, model FHS16, Kleve, Germany).

Nutrient digestibility was determined by detecting TiO2 in feed and fecal samples following the methods described by Short et al. [22] and was analyzed with a spectrometer (Synergy™ HTX Multi-Mode Microplate Reader, Biotek, Winooski, VT, USA). Apparent total tract digestibility (ATTD) of dry matter, gross energy, nitrogen, neutral detergent fiber (NDF), acid detergent fiber (ADF), and minerals were calculated as follows:

ATTD (%) = [1 − (Nutrient_feces_/Nutrient_diet_) × (TiO_2diet_/TiO_2feces_)] × 100

Nutrient_feces_ and nutrient_diet_ referred to the nutrient concentration in fecal and diet sample dry matter, while TiO_2diet_ and TiO_2feces_ indicated the concentration of TiO_2_ in diet and fecal samples.

##### Statistical Analysis

Growth performance data were analyzed using the Mixed procedure of SAS 9.3 (SAS Institute, Inc., Cary, NC, USA). Dietary treatments were the lone fixed effect and blocks based on the initial BW were the random effect. The pen served as the experimental unit for ANOVA. The levels of sodium butyrate were used in the interactive matrix language procedure of SAS to generate coefficients for orthogonal contrast for treatment BA (basal diet + 0.5% benzoic acid), SBA0.035 (BA + 0.035% sodium butyrate), SBA0.070 (BA + 0.070% sodium butyrate), and SBA0.105 (BA + 0.105% sodium butyrate). Orthogonal contrasts were also used to determine linear and quadratic effects of various levels of butyrate on growth performance. A contrast statement was also used to compare the difference between benzoic acid alone (BA) and NC. The probability value of *p* < 0.05 was considered significant and 0.05 < *p* < 0.10 was considered a statistical trend.

Nutrient digestibility and fecal volatile fatty acid (VFA) data were analyzed using the Mixed procedure of SAS 9.3 (SAS Institute, Inc., Cary, NC, USA) with such treatments as the main effect and initial BW blocks as the random effect. The pen was used as the experimental unit for nutrient digestibility and fecal VFA analysis. For blood parameters, data were analyzed using the repeated measure analysis with the Mixed procedure of SAS 9.3. The day post-weaning was the main factor in the repeated statement and the LSMEANS statement was used to compare the means of treatment, day, and treatment x day interaction with the Student’s t-test. The probability value of *p* < 0.05 was considered significant and 0.05 < *p* < 0.10 was considered a statistical trend.

##### DNA Extraction, Sequencing, and Microbiome Data Analysis

An in-depth and longitudinal analysis using next-generation sequencing was performed to provide insight into the diverse and complex gut microbiota, which allowed us to better understand how the gut microbiota of nursery pigs evolves under the influence of dietary acidifiers over time. Total DNA containing fecal microbial communities was extracted from individual fecal swab samples using the DNeasy PowerLyzer PowerSoil Kit (Qiagen, Germantown, MD, USA) according to the manufacturer’s protocol. DNA quantity was measured using NanoDrop One (Thermo Fisher Scientific, Madison, WI, USA) and diluted to 10 ng/μL.

PCR primers that flanked the V4 region of the bacterial 16S rRNA gene consisted of the Illumina adapter, an 8-nt index sequence, a 10-nt pad sequence, a 2-nt linker, and the gene-specific primer [23]. The gene-specific primer sequences were 5′-GTGCCAGCMGCCGCGGTAA-3′ (forward) and 5′-GGACTACHVGGGTWTCTAAT-3′ (reverse). The PCR products were electrophoresed on a 1% agarose gel to verify the size of amplicons and then purified using normalization plates (SequalPrep Normalization Plate Kit (Invitrogen, Carlsbad, CA, USA)). PCR amplicons purified from this system were pooled together to generate a sequencing library. In addition, the concentration and quality of the library were determined by KAPA Illumina Library Quantification Kits (Roche, Indianapolis, IN, USA) and an Agilent 2100 Bioanalyzer (Agilent, Santa Clara, CA, USA), respectively. Finally, the library was sequenced on a MiSeq sequencer (MiSeq Reagent Kit v2, 500 cycles (Illumina, San Diego, CA, USA)). A mock community (ZymoBIOMICS™ Microbial Community Standard (Zymo, Irvine, CA, USA)) was included in the sequencing run for quality control to estimate errors introduced during PCR amplification and the MiSeq run.

The 2 × 250 paired-end fastq files generated by the Miseq system were used as input files. Sequences were pre-processed, quality filtered (Q > 30), and analyzed using the QIIME2 (2019.10 release) platform [24]. Deblur [25] integrated with QIIME2 was used for sequence length trimming, denoising, chimera removal, and features binning at the single-nucleotide level. Naive Bayes classifier was used for assignment of our sequences into bacterial taxonomy using the Greengenes (v13_8 clustered at 99% identity) reference database, which was trimmed to contain only the V4 hypervariable region [26,27].

## 3. Results

### 3.1. The Effects of Organic Acids on Swine Growth Performance

#### 3.1.1. Trial #1: University of Illinois

**Figure 2:**
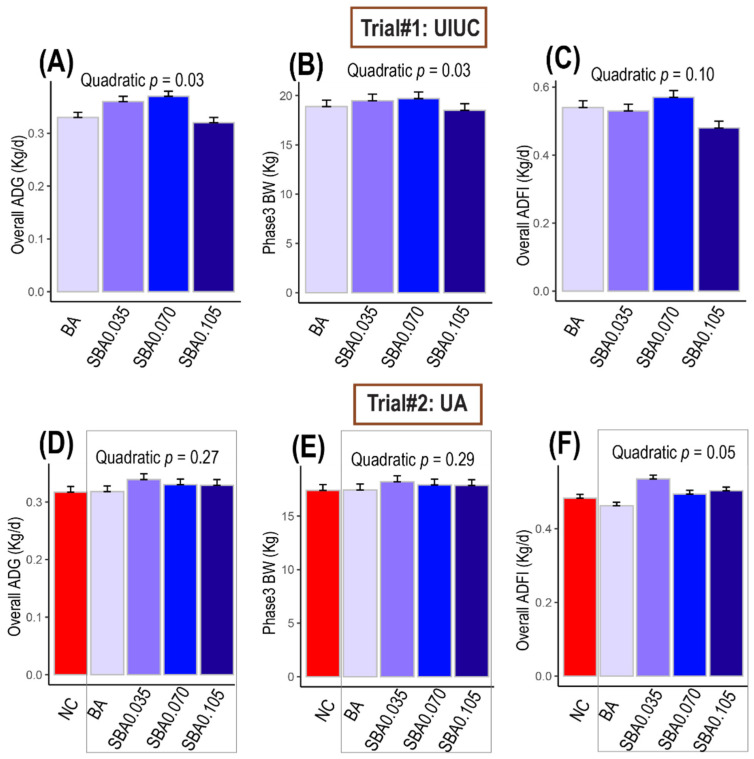
Effects of different doses of sodium butyrate (0%, 0.035%, 0.070%, and 0.105%) with 0.5% benzoic acid on (**A**) the average daily gain (ADG, kg/d), (**B**) body weight (BW, kg), and (**C**) the average daily feed intake (ADFI, kg/d) for the overall study at the University of Illinois at Urbana-Champaign; effects of the basal diet and different doses of sodium butyrate (0%, 0.035%, 0.070%, and 0.105%) with 0.5% benzoic acid on (**D**) the average daily gain (ADG, kg/d), (**E**) body weight (BW, kg), and (**F**) the average daily feed intake (ADFI, kg/d) for the overall study at the University of Arkansas. The data were analyzed by ANOVA using the PROC MIXED procedure in SAS in a completely randomized design with a pen as the experimental unit. Orthogonal contrasts were used to determine the linear and quadratic effects of various levels of butyrate on growth performance. In this figure, 0.1 ≤ *p* < 0.05 indicates a tendency for quadratic response to increasing levels of butyrate, *p* ≤ 0.05 indicates that the quadratic response to increasing levels of butyrate is significant (grey rectangle). BA: basal diet + 0.5% benzoic acid; SBA0.035: BA + 0.035% butyrate; SBA0.070: BA + 0.070% butyrate; SBA0.105: BA + 0.105% butyrate.

No differences in ADG (average daily gain), BW (body weight), ADFI (average daily feed intake), and G:F (gain/feed ratio) were observed in phase 1 (Tables S1 and S2). However, in phase 2, ADG and BW increased in the pigs fed diets supplemented with 0.035% (SBA0.035) or 0.070% butyrate (SBA0.070) and returned to the baseline in the pigs fed 0.105% butyrate diet (SBA0.105) compared with pigs fed 0.5% benzoic acid alone (BA; quadratic effect, *p* = 0.05). The same quadratic pattern was observed in phase 3 for ADG (*p* = 0.05; Table S1) and BW (Figure 2B, *p* = 0.03) and again for the ADG (Figure 2A) and G:F (Table S2) for the overall study (*p* < 0.03). A tendency for a quadratic increase in ADFI for the overall study was observed in pigs fed increasing levels of butyrate (Figure 2C, *p* = 0.10).

#### 3.1.2. Trial #2: University of Arkansas

Pigs fed the NC diet lost less weight, had higher feed intake and better G:F than pigs fed 0.5% benzoic acid in phase 1 (Tables S3 and S4; *p* = 0.01). At the end of phase 2, BW tended to be higher for pigs fed the NC diet compared with those fed BA (*p* = 0.06), and ADG and G:F were not different between these two treatments (*p* = 0.27 and *p* = 0.75, respectively).

Increasing butyrate in addition to 0.5% benzoic acid (BA) linearly increased ADG (*p* = 0.04 and *p* = 0.08) and BW in phases 1 and 2 (*p* = 0.03 and *p* = 0.02, respectively). Feed intake (ADFI) and G:F decreased with increasing butyrate in phase 1 (quadratic effect, *p* < 0.05), but ADFI and G:F were not different among treatments in phase 2 (Tables S3 and S4). For the overall study, the improvement in overall ADG (Figure 2D) and final BW at the end of phase 3 (Figure 2E) with moderate levels of butyrate when compared with pigs fed 0.5% benzoic acid alone were in agreement with results of Trial #1, although the differences were not significant (Table S3). Similarly, the overall ADFI was greater in pigs fed the 0.035% butyrate (SBA0.035) diet than in those fed 0.5% benzoic acid alone (BA; Figure 2F), then the feed intake declined in the pigs fed the diets containing 0.070% (SBA0.07) and 0.105% butyrate (SBA0.105; quadratic effect, *p* = 0.05). The overall G:F was not different among treatments (quadratic effect, *p* = 0.28).

The impact of acidifiers on the complete blood count (CBC) is summarized in Table S5. Pigs fed 0.5% benzoic acid alone (BA) tended to have a greater absolute monocyte count (*p* = 0.07) and percentage of monocytes over white blood cells (WBC; *p* = 0.09) than the NC-fed pigs. Additionally, pigs fed BA had a greater mean corpuscular volume (MCV; *p* = 0.01), mean corpuscular hemoglobin (MCH; *p* = 0.01), and mean corpuscular hemoglobin concentration (MCHC; *p* = 0.04) than the NC-fed pigs. A tendency for a quadratic response was observed in WBC (*p* = 0.07), neutrophil (*p* = 0.11), and eosinophil (*p* = 0.08) concentrations when pigs were fed increasing levels of butyrate, whereas a linear reduction was observed in lymphocyte (*p* = 0.09), MCHC (*p* = 0.03), and platelet (*p* = 0.02) concentration.

The total volatile fatty acid (VFA) contained in fecal samples (Table S6) from pigs fed 0.5% benzoic acid alone (BA) were not different from VFA in feces from pigs fed the NC diet (*p* = 0.58). A tendency for a quadratic increase in absolute butyrate (*p* = 0.07) and total VFA (*p* = 0.08) was observed from pigs fed increasing levels of butyrate with the greatest concentration observed in the pigs fed SBA0.070.

Pigs fed BA alone had greater digestibility of dietary dry matter, energy, nitrogen, ash, neutral detergent fiber (NDF), acid detergent fiber (ADF), and phosphorous than pigs fed the NC diet (Table 2; *p* < 0.01). Increasing levels of supplemental butyrate in pig diets reduced the digestibility of nitrogen (linear effect, *p* < 0.01), ash (quadratic effect, *p* < 0.01) and phosphorus (linear effect, *p* < 0.01).

### 3.2. Effects of Organic Acids on Swine Gut Microbiota

#### 3.2.1. DNA Sequence Data and Quality Control

We sequenced the V4 region of the 16S rRNA gene from a total of 360 fecal swab samples (n = 18/group, four timepoints). After filtering to remove low-quality sequences, 7,219,419 high-quality reads were obtained; the average number of sequencing reads generated per pig was 20,336 from a range of 6621 to 133,941. After the denoising step using Deblur, reads were clustered into 3044 operational taxonomic units at 100% identity; reads of each sample were rarefied at 5210 to address differences in library size for subsequent analysis.

#### 3.2.2. The Influence of Organic Acid Treatments on Gut Microbial Diversity

**Figure 3:**
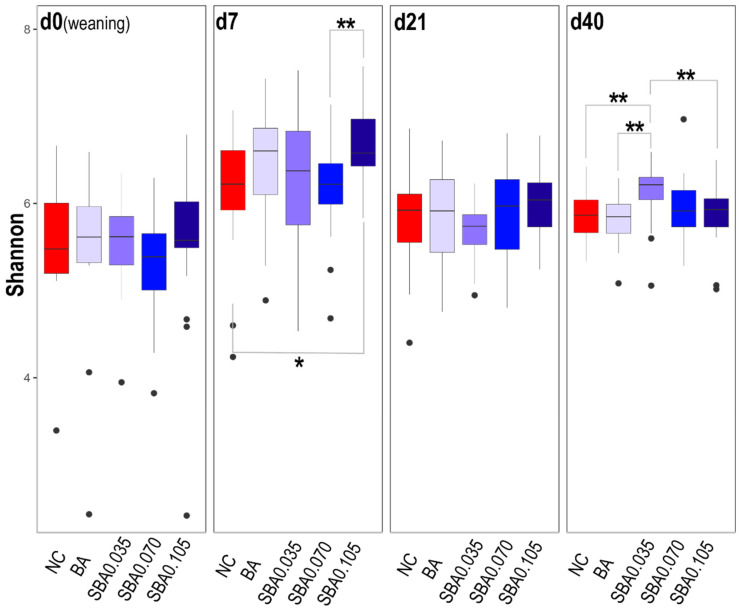
Alpha diversity for different dietary supplements at four timepoints. A common indicator (Shannon index) was used to measure bacterial diversity in all the groups. An asterisk (*) indicates a tendency for treatments to be significantly different (0.1 < *p* < 0.05); ** *p* < 0.05 indicates treatments are significantly different. NC: basal diet; BA: basal diet + 0.5% benzoic acid; SBA0.035: BA + 0.035% butyrate; SBA0.070: BA + 0.070% butyrate; SBA0.105: BA + 0.105% butyrate. Outliers are displayed as black dots.

The alpha diversity of samples from the pigs fed organic acid-free diets (NC) or benzoic acid diets supplemented with 0% (BA), 0.035% (SBA0.035), 0.070% (SBA0.070), and 0.105% butyrate (SBA0.105) was calculated using the Shannon index (Figure 3) and the observed features (Figure S1). The Shannon index, which accounts for both richness and evenness of a community, showed that pigs in the SBA0.105 group harbored a higher diversity of microbial species than the NC (*p* = 0.07) and SBA0.070 (*p* = 0.03) on d 7, whereas this greater microbial diversity disappeared on d 21. More interestingly, the microbiota in SBA0.035 pigs was more diverse than that in the NC, BA, and SBA0.105 groups (*p* = 0.05, *p* = 0.03, and *p* = 0.04, respectively) at the end of the nursery study (d 40), which indicated that long-term consumption of SBA0.035 may influence gut microbial diversity.

**Figure 4:**
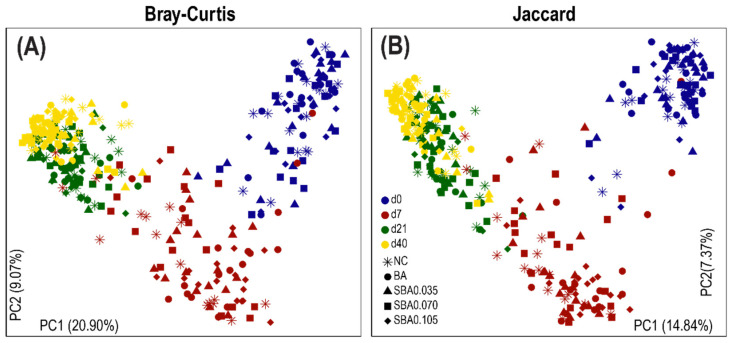
Longitudinal changes in the swine gut microbiome structure at four different timepoints. Principal coordinates analysis (PCoA) plots based on (**A**) the Bray–Curtis dissimilarity and (**B**) the Jaccard distances show distinct clusters. Colors blue, red, green, and yellow are used to differentiate between d 0 (weaning), d 7, d 21, and d 40, respectively. NC, BA, SBA0.035, SBA0.070, and SBA0.105 groups are differentiated by shapes (asterisk, circle, triangle, square, and diamond, respectively). NC: basal diet; BA: basal diet + 0.5% benzoic acid; SBA0.035: BA + 0.035% butyrate; SBA0.070: BA + 0.070% butyrate; SBA0.105: BA + 0.105% butyrate.

The principal coordinates analysis (PCoA) based on the Bray–Curtis dissimilarity showed that the overall structure of the gut microbiota significantly shifted from d 0 to d 40 in all the groups (Figure 4A). The analysis of similarities (ANOSIM) confirmed this pattern (Table 3) and d 0 samples which were distinctly different from those of the other three timepoints (d 7, d 21, and d 40). The swine gut microbiomes were different between d 7, d 21, and d 40 when pigs were fed solid diets; however, they were more similar to each other than to d 0 (weaning day) when the pigs had not yet consumed solid feed. Jaccard distances (Figure 4B) demonstrated the same pattern of the swine gut microbiota.

**Figure 5:**
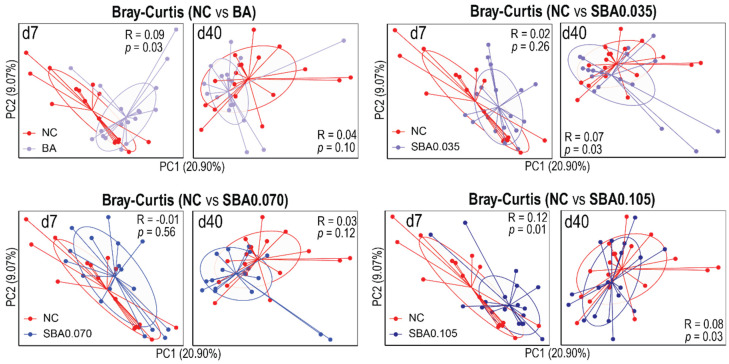
Beta diversity differences between NC and organic acid treatments. Principal coordinates analysis based on the Bray–Curtis dissimilarity revealed that a certain concentration of the organic acid blender impacted the bacterial structure during a specific window of the nursery stage. The analysis of similarity (ANOSIM) was used to determine the dissimilarity between NC and organic acid treatments. Samples are colored by groups. NC: basal diet; BA: basal diet + 0.5% benzoic acid; SBA0.035: BA + 0.035% butyrate; SBA0.070: BA + 0.070% butyrate; SBA0.105: BA + 0.105% butyrate.

In addition, we used Bray–Curtis dissimilarity to assess the effects of increasing doses of sodium butyrate (0%, 0.035%, 0.070%, and 0.105%) in combination with benzoic acid, as compared with a control diet (NC), on the gut microbiota community structure at two different timepoints (Figure 5). At the end of phase 1 (d 7), dietary treatments with benzoic acid alone or combined with low levels of sodium butyrate (BA, SBA0.035, and SBA0.070) had no or minimal effects on the structure of the gut microbiota (ANOSIM; BA vs. NC: R = 0.09, *p* = 0.03; SBA0.035 vs. NC: R = 0.02, *p* = 0.26; SBA0.070 vs. NC: R = −0.01, *p* = 0.56), whereas the highest level of sodium butyrate (SBA0.105) imposed noticeable effects on the gut microbiota (ANOSIM; SBA0.105 vs. NC: R = 0.12, *p* = 0.01). Although swine gut microbiota susceptibility to SBA0.105 was reduced as pigs got older (d 40 ANOSIM; SBA0.105 vs. NC: R = 0.08, *p* = 0.03), SBA0.105 still had the greatest impact on the gut bacteria structure when compared to other treatments (d 40 ANOSIM; BA vs. NC: R = 0.04, *p* = 0.10; SBA0.035 vs. NC: R = 0.07, *p* = 0.03; SBA0.070 vs. NC: R = 0.03, *p* = 0.12). Consistent with our findings on the alpha diversity, the effect of SBA0.105 on the gut microbiota community structure also disappeared on d 21 (Figure S2; ANOSIM; SBA0.105 vs. NC: R = −0.10, *p* = 0.6). The other treatments had no effects on the gut microbiota structure either (Figure S2; ANOSIM; BA vs. NC: R = −0.02, *p* = 0.70; SBA0.035 vs. NC: R = 0.05, *p* = 0.1; SBA0.070 vs. NC: R = −0.02, *p* = 0.70).

#### 3.2.3. Gut Microbiota Composition in Response to the Use of Organic Acids

**Figure 6:**
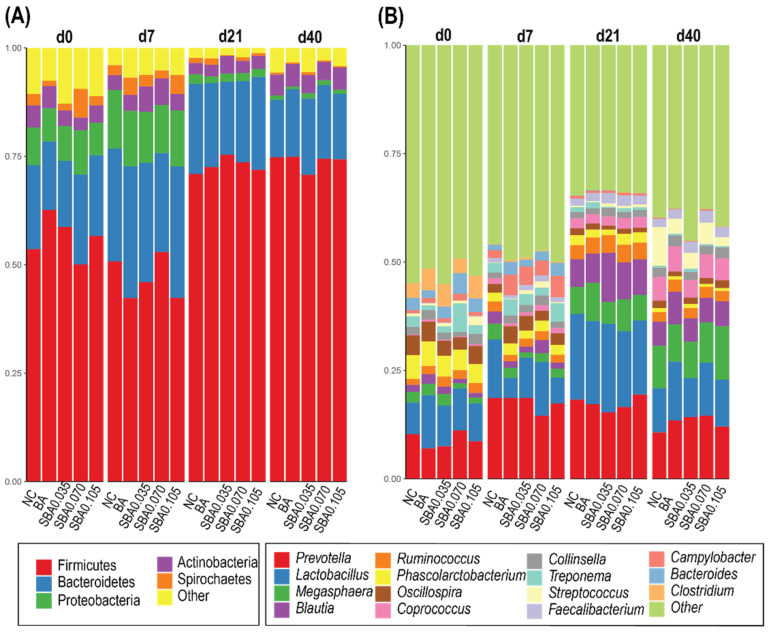
Relative bacterial abundance for each treatment at different timepoints. The relative abundance of (**A**) top 5 phyla and (**B**) top 15 genus-classified rectal microbiomes at d 0 (weaning), d 7, d 21, and d 40 is reported. NC: basal diet; BA: basal diet + 0.5% benzoic acid; SBA0.035: BA + 0.035% butyrate; SBA0.070: BA + 0.070% butyrate; SBA0.105: BA + 0.105% butyrate.

Data from pigs of the same treatment and sampling date were grouped to evaluate the organic acid mixture effects on the gut microbial community. The fecal microbiota composition for the animals receiving different diets and how it changed over time are shown in Figure 6A. Two phyla, Firmicutes and Bacteroidetes, were the most dominant in the fecal samples regardless of age or treatment group, and they comprised up to 90% of the total sequences on d 21 and d 40. The proportion of bacteria in the phyla Firmicutes increased as the pigs got older, whereas the proportion of bacteria in the phyla Bacteroidetes and Proteobacteria decreased.

The relative abundances of features at the genus level are shown in Figure 6B. For each treatment, the most represented genera at all timepoints were *Lactobacillus* and *Prevotella*. The increase in the relative abundance of *Prevotella* in each treatment was particularly striking after introducing solid feed: from 8.9% of the population on the weaning day (d 0) to 17.6% of the population on d 7 post-weaning. The relative abundances of *Megasphaera* and *Blautia* also increased after weaning. The subdominant gut microbiota component varied at different timepoints. *Phascolarctobacterium*, an important subdominant component of the gut microbiota on d 0 (weaning day), decreased significantly on the following timepoints. *Faecalibacterium* appeared on d 7 and persisted until the end of the nursery phase; however, the largely enriched *Campylobacter* and *Bacteroides* on d 7 were almost absent on d 21 and d 40. Moreover, *Streptococcus* dramatically increased on d 40.

#### 3.2.4. Linear Discriminant Analysis of Gut Microbiota

**Figure 7:**
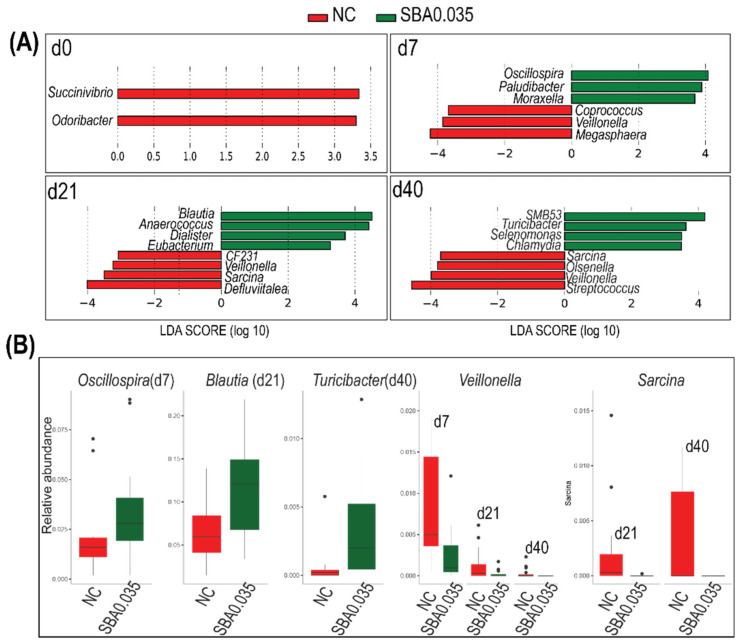
LEfSe analysis of the swine gut microbiome data. (**A**) Differentially abundant genera between NC and SBA0.035 piglets shown by sampling time. The genera in this graph were statistically significant (*p* < 0.05) and had an LDA Score > 2. (**B**) Relative abundance of the important genera selected by LEfSe, outliers are displayed as black dots. NC: basal diet; SBA0.035: NC + 0.5% benzoic acid +0.035% butyrate.

To further investigate how the composition of fecal bacteria changed in the 0.035% butyrate (SBA0.035) group, which revealed a high degree of bacterial diversity and considerable growth performance at the end of the nursery study, a linear discriminant analysis Effect Size (LEfSe) analysis was performed to determine the most differentially abundant genera between NC (devoid of organic acids) and SBA0.035. At four timepoints, 25 differentially represented taxa at the genus level were identified (Figure 7A). The results showed that some of the biomarker genera like *Oscillospira* (d 7), *Blautia* (d 21), and *Turicibacter* (d 40) were significantly more abundant in the 0.035% butyrate group (SBA0.035), whereas *Veillonella* (d 7, d 21, d 40) and *Sarcina* (d 21 and d 40) were more abundant in the NC group (Figure 7B). The LEfSe analysis was also used to identify the difference between NC and other organic acid groups. We found all organic acid groups can decrease the abundance of *Veillonella* and *Sarcina*. In addition, both benzoic acid alone (BA) and SBA0.105 increased the population of *Oscillospira* on d7, and SBA0.070 increased the relative abundance of *Turicibacter* on d 40 (Figures S3–S5).

#### 3.2.5. The Signature Microbiome-Differentiating Organic Acid Supplementary

**Figure 8:**
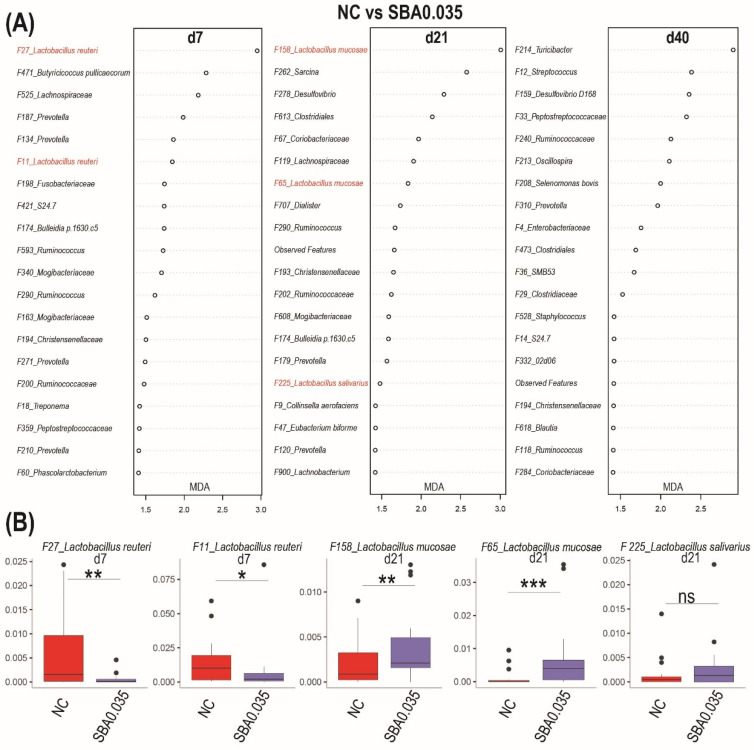
Gut microbiome signature of the organic acid cocktail. (**A**) Top 20 most predictive features that differentiate SBA0.035 from NC at three timepoints. These features were ranked by a random forest based on their important score (MDA: mean decrease accuracy). (**B**) Relative abundance of features labeled in red in the panel. Outliers are displayed as black dots. An asterisk (*) indicates a tendency for treatments to be significantly different (0.1 < *p* < 0.05), ** *p* < 0.05, and *** *p* < 0.01 indicates treatments that are significantly different, ns indicates no difference. NC: basal diet; SBA0.035: NC + 0.5% benzoic acid +0.035% butyrate.

We next used a random forest to identify microbial signatures that best differentiate the NC and SBA0.035 groups at the species level. We included alpha diversity measures (Shannon index and observed features) and the relative abundance of the top 500 bacterial features of each phase in the random forest model. The top 20 bacterial features that predicted treatment at each phase are listed in Figure 8A. Surprisingly, the SBA0.035 treatment decreased the relative abundance of some potential beneficial bacteria, such as *Lactobacillus reuteri* (F11, F27) at the end of phase 1 (d 7; Figure 8B). However, the continued use of organic acid blender SBA0.035 in the animal feed increased the number of *Lactobacillus* species (F65, F158, and F225).

## 4. Discussion

Environmental conditions, animal management practices, genetic background, and health status greatly impact the repeatability of animal trials. Therefore, a multiple-station study was used as an attempt to provide more conclusive findings from this experiment. This experiment involved two research stations and aimed to evaluate the optimum level of sodium butyrate in a diet containing 0.5% benzoic acid on the growth performance of weanling pigs under different conditions.

During the early post-weaning period, pig intestines undergo reconstruction to accommodate the change to a solid diet. Furthermore, during this period, psychological stress brings about lower feed intake resulting in the potential for nutritional deficiencies. Commensal bacteria are unable to achieve their optimal number and full functional capacity. Butyrate, a microbiota-produced short-chain fatty acid, plays an important role between the microbiota and the immune system [28]. Butyrate reduces the pro-inflammatory response induced by allergic reactions (mediated by type 2 innate lymphoid cells) and pathogen-associated molecular patterns (oriented via the toll-like receptor signaling pathway) [29,30,31]. Butyrate is also a source of energy for intestinal development and repair [14,32]. Thus, the addition of butyrate to the diet could greatly improve intestinal health, such as regulating intestinal epithelial cell proliferation, balancing the gut microbiota, and developing intestinal mucosa immunity. Our findings are consistent with the findings by Piva et al. (2002) who reported a superior weight gain and feed intake in the pigs fed butyrate. However, this beneficial effect of butyrate was appreciable only in the first two weeks post-weaning and was diminished in subsequent growth periods [33]. To further extend the beneficial effects of butyrate on growth performance from early weaning to the following stages, we added 0.5% benzoic acid with increasing levels of sodium butyrate (0%, 0.035%, 0.070%, 0.105%) at both stations. Benzoic acid has been recognized as an antimicrobial agent with a broad spectrum of activity against pathogenic fungi and bacteria [34] and has been commonly used as a preservative for foods and beverages [35,36]. Moreover, evidence suggested that benzoic acid increases digestive enzyme activity and improves jejunal and ileum morphology leading to greater nutrient digestibility in both poultry and swine [37,38]. This increase in nutrient uptake not only reduces nonpathogenic diarrhea, but also improves the growth performance of nursery pigs [39]. In order to determine the impact of benzoic acid alone or in combination with butyrate on nutrient digestibility, a negative control (basal diet) group was added at the University of Arkansas. Interestingly, we found that digestibility of dry matter, energy, nitrogen, ash, neutral- and acid-detergent fiber, and phosphorous increased in the pigs fed benzoic acid alone (BA), which is consistent with the higher numerical G:F observed in phase 3 and could be due to higher digestive enzyme activity [37,40]. The present study clearly demonstrated that benzoic acid further extended the benefits of butyrate into the late nursery period, which suggests synergistic effects existed between butyrate and benzoic acid. At the same time, results indicated that sodium butyrate included at 0.035% or 0.07% in the diet containing 0.5% benzoic acid resulted in better growth performance compared with the other treatments. However, adding increasing concentrations of butyrate into the BA diet gradually decreased nutrient digestibility to the levels at or below that observed in the NC-fed pigs. The reason for this outcome is unclear, and further research is needed.

The intestinal microbiota assists the host in energy absorption, epithelium development, immune system enhancement, pathogen inhibition, and the fermentation of non-digestible foods to short-chain fatty acids (SCFA) and other metabolites [41,42]. In the present study, Firmicutes and Bacteroidetes were the two most predominant phyla in the piglet gut microbiota, which was consistent with our previous piglet studies [43,44,45,46]. Similarly, as in our previous reports, *Prevotella* and *Lactobacillus* were the abundant genera in the present study. The relative abundance of *Prevotella* increased remarkably after solid feed was introduced. This change might be associated with diet variation, since nursery pigs faced a dietary transition from sow milk to corn/soybean meal-based diets. Many studies have shown that high abundance of *Prevotella* is associated with plant-based foods. For example, De Filippo found *Prevotella* was exclusively present in the children consuming a traditional rural African diet rich in starch, fiber, and plant protein compared to the children eating a typical Western diet high in animal protein, sugar, starch, and fat and low in fiber [47]. Like *Prevotella* in the swine gut, *Megasphaera* and *Blautia* also increased during the post-weaning period. These two bacteria are involved in the digestion of the carbohydrates in the daily diet [48,49,50]. Taken together, data indicated that the gut microbiota might coevolve with the diet of pigs, allowing them to better degrade plant carbohydrate diets and increase nutrient uptake, thus benefitting the host. Furthermore, the swine gut microbiome presented certain anti-inflammatory bacteria at each timepoint, such as *Bacteroides* and *Faecalibacterium* [51,52]. This could indicate that maintaining an intestinal microbiota with a potentially anti-inflammatory function is important throughout all life stages.

High gut alpha diversity has always been linked to a healthy status in many human studies [53,54]. Our data revealed that greater overall diversity was an indicator of increased growth performance in pigs. For example, the SBA0.035 group had greater alpha diversity than those of other groups except for the SBA0.070 group at the end of the study, which was associated with better growth performance. However, high bacterial diversity temporarily appeared in the SBA0.105 group on d 7 and disappeared at the following timepoints; this temporary high diversity did not correlate with improved growth performance. It is possible that newly-weaned pigs had a limited capacity to maintain low gastric pH and their gastrointestinal bacteria were more vulnerable to changes; thus, the highest concentration of acidifier (SBA0.105) could easily cause temporary changes in bacterial diversity during the first week after weaning. In addition, a drop in growth performance is commonly observed in post-weaning pigs due to stress.

Supplementing SBA0.035 significantly increased the relative abundance of *Oscillospira* on d 7. *Oscillospira* is an anaerobic bacterial genus from *Clostridium* cluster IV belonging to the Firmicutes phylum. It is a common and abundant member of the human gut microbiota and is recognized as a member of the core microbiota related to health [55,56]. Some *Oscillospira* species might be butyrate producers, such as *O. ruminantium* [57,58]. Numerous recent studies have indicated that the fecal *Oscillospira* level is reduced during inflammatory diseases. For example, results of several meta-analyses of microbiota studies demonstrated that *Oscillospira* was significantly reduced in patients with Crohn’s disease [59], which is a chronic inflammatory bowel disease characterized by intestinal disorders, causing abdominal pain, severe diarrhea, weight loss, and malnutrition. The relative abundance of *Oscillospira* also showed a reduction in pediatric nonalcoholic steatohepatitis [60], an inflammatory liver disease characterized by a buildup of fat in the liver. Based on its negative association with inflammatory diseases, we speculated that more abundant *Oscillospira* might benefit the host. Although the high level of *Oscillospira* in the SBA0.035 group did not improve growth performance in nursery phase 1, it may have helped balance the intestinal bacteria and lead the way to a healthier pig gut. Notably, the relative abundance of *Oscillospira* could be affected by short-term dietary interventions. This is confirmed by a recent study where the relative *Oscillospira* abundance greatly increased with the switch to an animal-based diet and decreased (more mildly) in the plant-based diet [61]. This could partly explain why the relative *Oscillospira* abundance gradually decreased when the diet converted from the milk-based one to the plant-based one.

As previously described in the results, the relative abundance of *Blautia* significantly increased in the feces of the SBA0.035 group on d 21. Species of *Blautia* are SCFA producers [62]. SCFAs are bacterial fermentation end products and are known to perform various beneficial functions in the gut, such as maintaining epithelial barrier integrity, regulating the immune system, exerting anti-inflammatory effects, providing the energy source for colonocytes, and regulating epithelial gene expression [63]. Therefore, the stimulation of SCFA production by *Blautia* could be useful for sustaining health and enhancing swine growth performance.

The increased relative abundance of *Turicibacter* in the SBA0.035 group on d 40 suggested a potential impact of the organic acid mixture on the swine immune system. It has been demonstrated that the relative abundance of *Turicibacter* was linked to host immunity and could serve as an indicator of a well-functioning immune system in mice. For example, immunodeficient mice harbored a lower relative abundance of *Turicibacter* compared to their wild type counterparts [64]. Furthermore, *Turicibacter* could help reduce susceptibility to *Salmonella*-induced inflammation in mice lacking B4galnt2 expression in the gut [65]. A previous study also showed that *Turicibacter* positively influenced swine growth performance [43]. Hence, *Turicibacter* might possess immunomodulatory characteristics in the swine gut microbiota, consequently promoting growth performance.

In our dataset, the genera *Veillonella* and *Sarcina* decreased significantly in all the organic acid groups compared with NC. *Veillonella* has been recently linked to various inflammatory diseases, such as primary sclerosing cholangitis and inflammatory bowel disease [66,67]. *Sarcina* is another harmful bacterial genus that can cause disease in both humans and animals. The presence of *Sarcina* may delay gastric emptying and cause a lethal gastric bloating-like syndrome in animals [68,69]. These data indicate that both *Veillonella* and *Sarcina* may be detrimental to swine growth. Thus, significantly decreasing the relative abundance of *Veillonella* and *Sarcina* by supplementing organic acids may reduce disease risk in swine.

There are some limitations to the study. First, we used fecal swab samples to represent the swine gut microbiota. The use of fecal rectal swabs is a non-invasive way to represent the gut microbiota for longitudinal studies that follow the same sets of animals without sacrifice [43,46]. However, these rectal swab samples may not necessarily reflect the microbiota of other sections due to the divergence of bacteria throughout the intestines [70]. Second, a sodium butyrate alone group should be included. Treatment with sodium butyrate alone would allow us to better distinguish the responses we observed from each organic acid. Third, nutrient digestibility in the current study only represented the response in phase 3. Nutrient digestibility in the early weaning period should have been examined. This would have helped determine if the effects of organic acids on nutrient digestibility are consistent throughout the nursery period. Finally, due to the limited funding and facilities, we were only able to examine the effect of organic acids on the swine gut microbiome and phenotypes till the end of the nursery stage. Further studies are desired to evaluate their effects on growth performance and gut microbiota during the growing and finishing stages.

## 5. Conclusions

In conclusion, results of the present research demonstrated that butyrate improved overall growth performance of the pigs fed diets containing 0.5% benzoic acid. This result may be due to these diets significantly increasing the variety and proportion of the putative beneficial bacteria and greatly reducing the bacteria that may be detrimental to pig health. Further research is warranted to determine if the gut microbiota shaped by organic acids early in life would lead to long-lasting beneficial effects on swine performance later in life. In addition, we found that varying the diet plays a crucial role in shaping the gut microbial community of piglets.

## Figures and Tables

**Table 1:** Experimental dietary composition for each phase.

	Trial #1			Trial #2		
Ingredients, %	Phase 1	Phase 2	Phase 3	Phase 1	Phase 2	Phase 3
Corn, yellow dense ^1^	29.02	31.42	49.53	29.90	32.18	49.88
Dried distillers grain with solubles (6–10% fat)	5.00	15.00	15.00	5.00	15.00	15.00
Dried whey	8.00	4.00	0.00	8.00	4.00	0.00
Soybean meal, 48% CP	22.65	28.05	29.30	22.65	28.05	29.30
Oats	15.00	12.50	0.00	15.00	12.50	0.00
Fish meal	5.00	3.15	0.00	5.00	3.15	0.00
Lactose	0.25	0.00	0.00	0.25	0.00	0.00
Enzymatic SBM	9.50	0.00	0.00	9.50	0.00	0.00
Soybean oil	2.50	2.50	2.50	2.50	2.50	2.50
Benzoic acid ^2^	0.50	0.50	0.50	0.50	0.50	0.50
Other ^3^	2.58	2.95	3.24	2.58	2.95	3.24
**Calculated**						
Metabolizable energy (kcal/kg)	3274	3233	3451	3455	3429	3402
Crude protein (%)	25.62	23.94	22.17	26.50	25.03	22.84
SID lysine (%)	1.50	1.35	1.23	1.46	1.42	1.28
Available P (%)	0.45	0.40	0.33	0.41	0.30	0.22
Ca (%)	0.85	0.80	0.70	0.76	0.66	0.56
**Analyzed**						
Gross energy (kcal/kg)	4145	4094	4086	4529	4584	4558
Crude protein, %	26.97	23.03	21.90	26.40	24.10	23.20

^1^ Sodium butyrate (Villimax^®^, DSM Nutritional Products Inc, Parsippany, NJ, USA) was supplemented at levels of 0%, 0.035%, 0.070%, 0.105% to generate BA, SBA0.035, SBA0.070, and SBA 0.105 treatment, respectively, in each phase. ^2^ Benzoic acid (Vevovitall^®^, DSM Nutritional Products Inc, Parsippany, NJ, USA): all the diets contained 0.5% benzoic acid except for the negative control diet at the University of Arkansas. ^3^ Other contained limestone, monocalcium phosphate, trace minerals premix, vitamin premix, feed-grade amino acids, and phytase. The vitamin premix provided the following per kg of complete diet: 397.5 mg of Ca as CaCO3, 11,022.9 IU of vitamin A, 1377.9 IU of vitamin D3, 44.09 IU of vitamin E, 0.0386 mg of vitamin B12, 4.41 mg of menadione, 8.27 mg of riboflavin, 27.56 mg of D-pantothenic acid, and 49.6 mg of niacin. The minerals premix provided the following per kg of complete diet: 84 mg of Ca as CaCO3, 165 mg of Fe as FeSO4, 165 mg of Zn as ZnSO4, 39.6 mg of Mn as MnSO4, 16.5 mg of Cu as CuSO4, 0.3 mg of I as CaI2, and 0.3 mg of Se as Na2SeO3. Note: diets were antibiotic-free and were formulated without pharmaceutical levels of zinc or copper.

**Table 2:** Effects of organic acids on the apparent total tract digestibility of nutrients.

		Treatment ^1^					*p*-Value		
	BA	SBA0.035	SBA0.070	SBA0.105	NC	SEM	Linear	Quadratic	BA vs. NC
DM ^2^	0.94	0.93	0.92	0.92	0.92	0.00	0.11	0.71	<0.01
Energy	0.84	0.79	0.78	0.78	0.78	0.01	0.17	0.51	<0.01
Nitrogen	0.81	0.76	0.72	0.70	0.72	0.02	<0.01	0.73	<0.01
Ash	0.65	0.55	0.46	0.50	0.52	0.02	0.08	<0.01	<0.01
NDF ^3^	0.73	0.65	0.62	0.62	0.63	0.02	0.15	0.47	<0.01
ADF ^4^	0.66	0.55	0.54	0.52	0.59	0.02	0.32	0.67	0.03
Phosphorus	0.58	0.49	0.37	0.36	0.39	0.03	<0.01	0.11	<0.01

^1^ NC: basal diet devoid of any added organic acid; BA: basal diet + 0.5% benzoic acid; SBA0.035: BA + 0.035% butyrate; SBA0.070: BA + 0.070% butyrate; SBA0.105: BA + 0.105% butyrate. ^2^ DM: dry matter. ^3^ NDF: neutral detergent fiber. ^4^ ADF: acid detergent fiber. Statistical significance and tendencies were considered at *p* < 0.05 and 0.05 ≤ *p* < 0.10, respectively.

**Table 3:** Dissimilarities in the swine gut microbiome at different timepoints.

Group 1	Group 2	Sample Size	Permutations	Bray–Curtis			Jaccard		
				R	*p*-Value	q-Value	R	*p*-Value	q-Value
d0	d7	176	999	0.69	0.001	0.001	0.80	0.001	0.001
d0	d21	179	999	0.82	0.001	0.001	0.91	0.001	0.001
d0	d40	178	999	0.84	0.001	0.001	0.92	0.001	0.001
d7	d21	177	999	0.56	0.001	0.001	0.68	0.001	0.001
d7	d40	176	999	0.69	0.001	0.001	0.83	0.001	0.001
d21	d40	179	999	0.46	0.001	0.001	0.49	0.001	0.001

The analysis of similarity (ANOSIM) based on the Bray–Curtis dissimilarity and the Jaccard distances was used to calculate the dissimilarities in the swine gut microbiome at different timepoints.

## Data Availability

Raw data were submitted to the National Center for Biotechnology Information (NCBI) Short Read Archive database and are available with BioProject accession number PRJNA689421.

## Supplementary Materials

The following are available online at https://www.mdpi.com/2076-2607/9/1/110/s1, Figure S1: Alpha diversity measure for different dietary supplements at four time points. Common indicator-Observed Features was used to measure bacterial diversity in all groups, Figure S2: Principal coordinate analysis based on Bray-Curtis distances was used to detect the effects of different concentrations of organic acid blender on bacterial structure on d 21, Figure S3: LEfSe analysis of the swine gut microbiome data between NC and BA groups, Figure S4: LEfSe analysis of the swine gut microbiome data between NC and SBA0.070 groups, Figure S5: LEfSe analysis of the swine gut microbiome data between NC and SBA0.105 groups, Table S1: Effects of organic acids on average daily gain and body weight of nursery pigs (Trial#1), Table S2: Effects of organic acids on average daily feed intake and feed efficiency of nursery pigs (Trial#1), Table S3: Effects of organic acids on average daily gain and body weight of nursery pigs (Trial#2), Table S4: Effects of organic acids on average daily feed intake and feed efficiency of nursery pigs (Trial#2), Table S5: Effects of organic acids on complete blood count of nursery pigs (Trial#2), Table S6: Effects of organic acids on volatile fatty acid concentration in fecal samples (Trial#2).
