# Supplementary material for: Dietary Organic Acids Modulate Gut Microbiota and Improve Growth Performance of Nursery Pigs
Source: Microorganisms. 2021 Jan 5;9(1):110. doi: 10.3390/microorganisms9010110 (PMC7824888; doi:10.3390/microorganisms9010110)
Supplement: Supplementary file 1 [file microorganisms-09-00110-s001.zip › Supplemental Files - 1.4new/Organic acid supplemental Figures.docx]

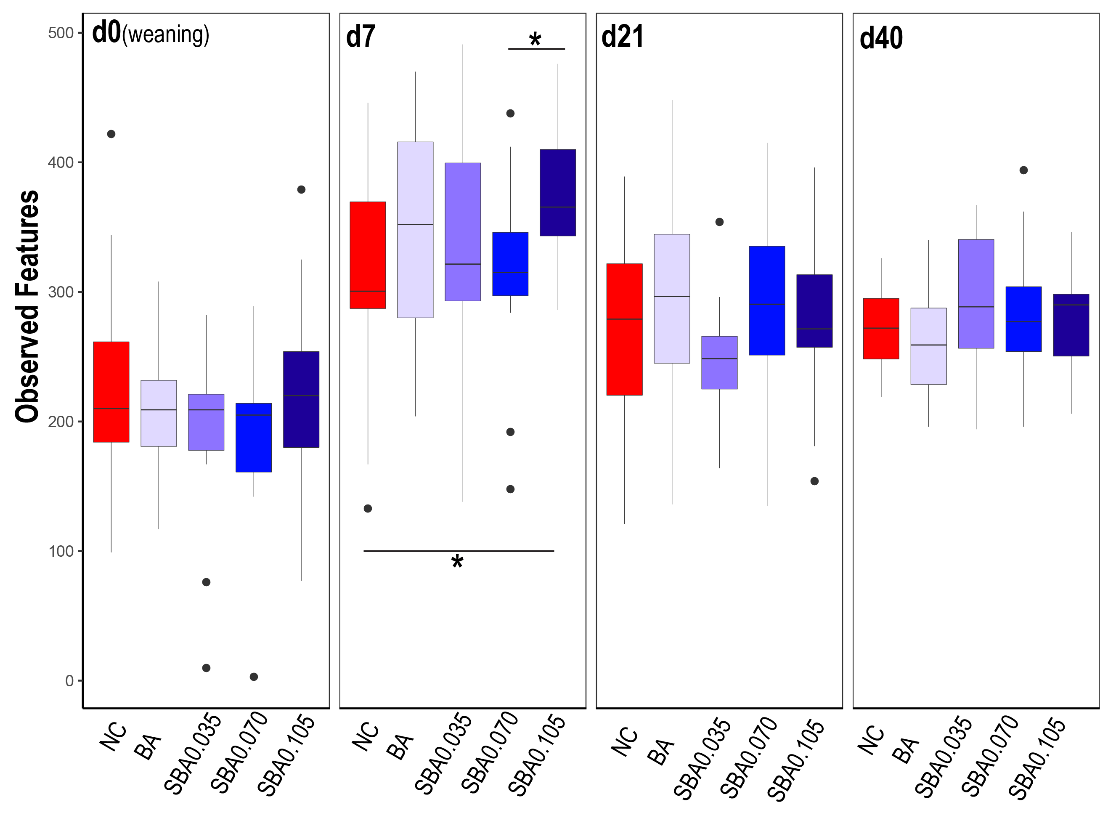


**Figure S1**. Alpha diversity measure for different dietary supplements at four time points. Common indicator-Observed Features was used to measure bacterial diversity in all groups. An asterisk (*) indicates a tendency for treatments significantly different (0.1 < *p* < 0.05). NC: basal diet; BA: basal diet+0.5% benzoic acid; SBA0.035: BA+0.035% butyrate; SBA0.070: BA+0.070% butyrate; SBA0.105: BA+0.105% butyrate. Outliers are displayed as black dots.


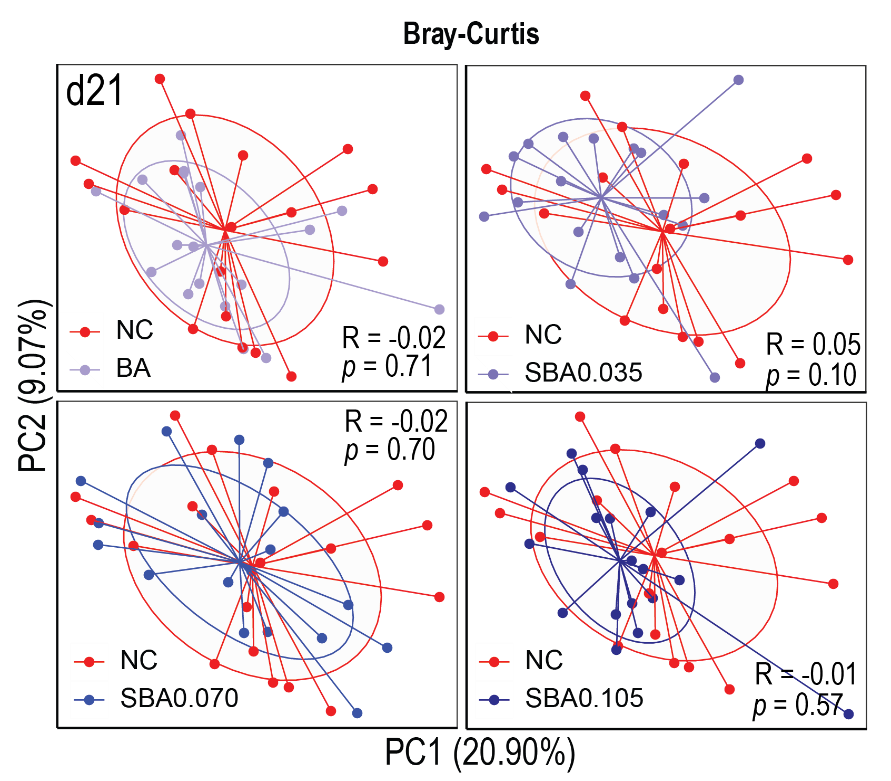


**Figure S2**. Principal coordinate analysis based on Bray-Curtis distances was used to detect the effects of different concentrations of organic acid blender on bacterial structure on d 21. Differences in beta diversity between NC and organic acid treatment were tested by the analysis of similarity (ANOSIM). Samples are colored by groups. NC: basal diet; BA: basal diet+0.5% benzoic acid; SBA0.035: BA+0.035% butyrate; SBA0.070: BA+0.070% butyrate; SBA0.105: BA+0.105% butyrate.


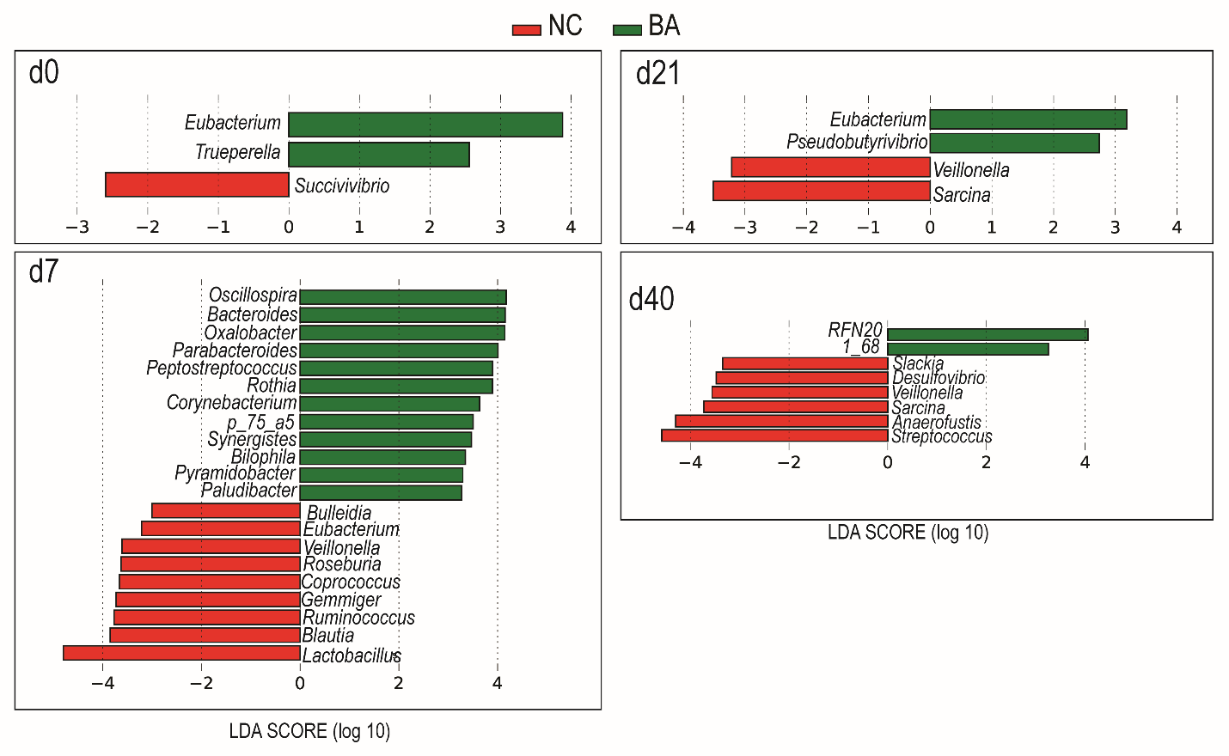


**Figure S3**. LEfSe analysis of the swine gut microbiome data between NC and BA groups. Differentially abundant genera between NC and BA piglets were shown by sampling time. Genera in this graph were statistically significant (*p* < 0.05) and had an LDA Score > 2. NC: basal diet; BA: NC+0.5% benzoic acid.


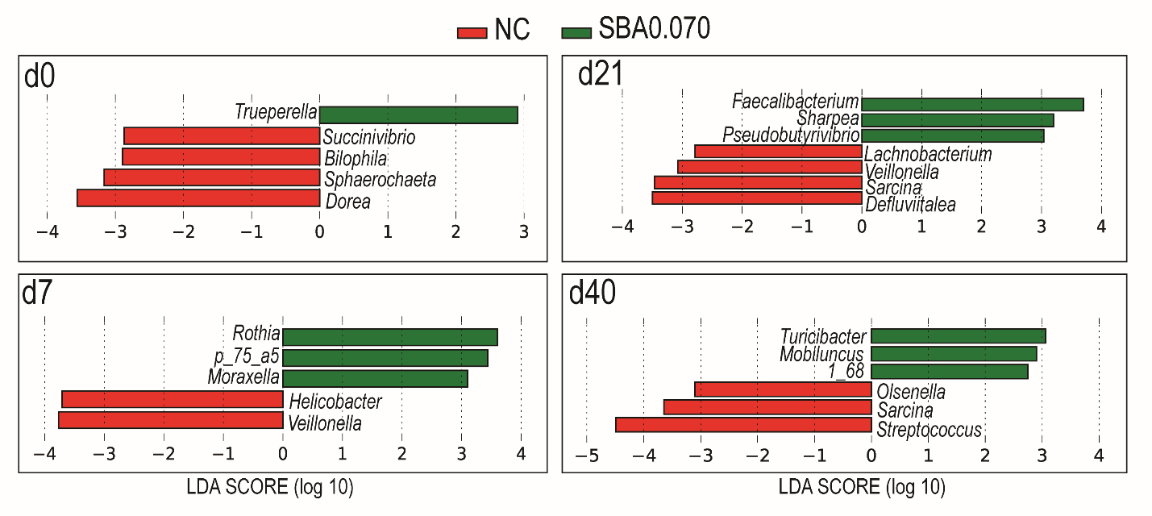


**Figure S4.** LEfSe analysis of the swine gut microbiome data between NC and SBA0.070 groups. Differentially abundant genera between NC and SBA0.070 piglets were shown by sampling time. Genera in this graph were statistically significant (*p* < 0.05) and had an LDA Score > 2. NC: basal diet; SBA0.070: NC+0.5% benzoic acid+0.070% butyrate.


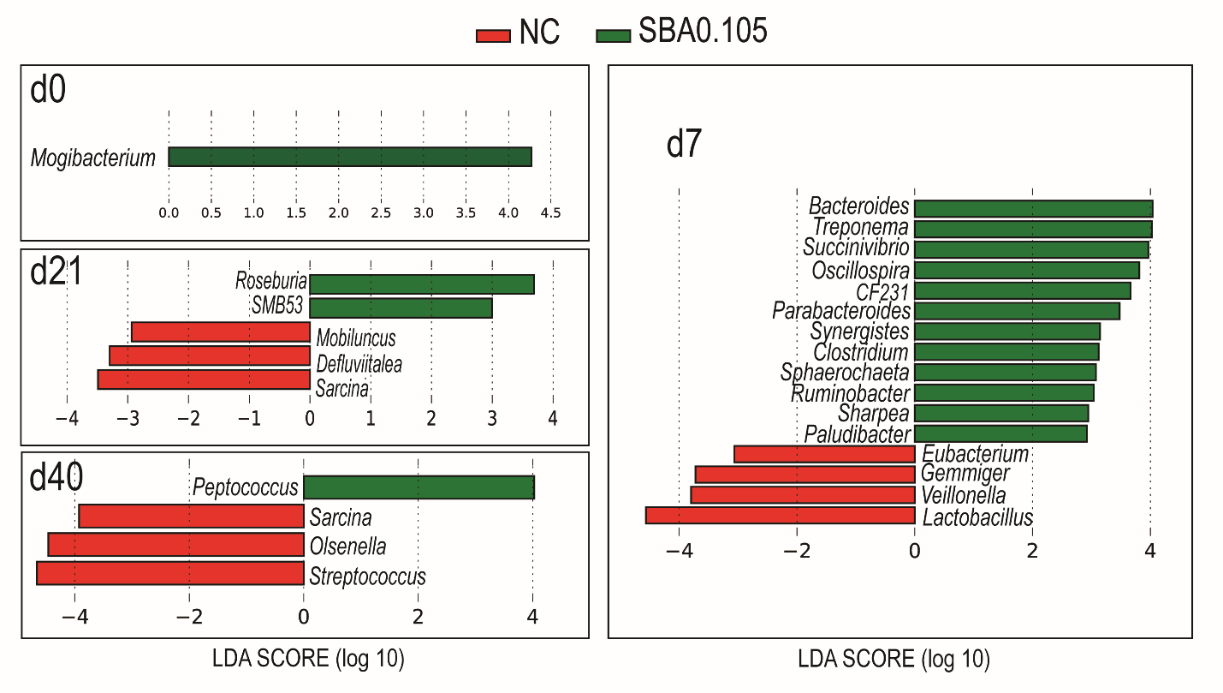


**Figure S5.** LEfSe analysis of the swine gut microbiome data between NC and SBA0.105 groups. Differentially abundant genera between NC and SBA0.105 piglets were shown by sampling time. Genera in this graph were statistically significant (*p* < 0.05) and had an LDA Score > 2. NC: basal diet; SBA0.105: NC+0.5% benzoic acid+0.105% butyrate.
