# Supplementary material for: Dietary Organic Acids Modulate Gut Microbiota and Improve Growth Performance of Nursery Pigs
Source: Microorganisms. 2021 Jan 5;9(1):110. doi: 10.3390/microorganisms9010110 (PMC7824888; doi:10.3390/microorganisms9010110)
Supplement: Supplementary file 1 [file microorganisms-09-00110-s001.zip › Supplemental Files - 1.4new/Organic acid supplemental Tables.docx]

**Supplemental Table S1**. Effects of organic acids on average daily gain and body weight of nursery pigs (Trial#1)

|  | Treatment | | | | |  | *p* value | | |
| --- | --- | --- | --- | --- | --- | --- | --- | --- | --- |
|  | BA | | SBA0.035 | SBA0.070 | SBA0.105 | SEM | Treatment | Linear | Quadratic |
| **ADG, kg/d** | |  |  |  |  |  |  |  |  |
| Phase1 | | 0.00 | 0.01 | 0.02 | 0.00 | 0.01 | 0.61 | 0.86 | 0.44 |
| Phase2 | | 0.25 | 0.31 | 0.30 | 0.25 | 0.02 | 0.08 | 0.93 | 0.05 |
| Phase3 | | 0.57 | 0.58 | 0.58 | 0.55 | 0.02 | 0.48 | 0.84 | 0.05 |
| Overall | | 0.33 | 0.36 | 0.37 | 0.32 | 0.01 | 0.04 | 0.71 | 0.03 |
| **BW, kg** | |  |  |  |  |  |  |  |  |
| Initial | | 6.89 | 6.81 | 6.92 | 6.89 | 0.21 | 0.99 | 0.88 | 0.37 |
| Phase1 | | 6.91 | 6.89 | 7.06 | 6.89 | 0.24 | 0.95 | 0.82 | 0.33 |
| Phase2 | | 10.17 | 11.13 | 11.01 | 10.15 | 0.41 | 0.17 | 0.89 | 0.05 |
| Phase3 | | 18.88 | 19.48 | 19.70 | 18.51 | 0.66 | 0.49 | 0.72 | 0.03 |
| ADG: average daily gain; BW: body weight; BA: basal diet+0.5% benzoic acid; SBA0.035: BA+0.035% butyrate; SBA0.070: BA+0.070% butyrate; SBA0.105: BA+0.105% butyrate. Phase1: d0 (weaning)-7; Phase2: d7-21; Phase3: d21-35; Overall: d0-35. Statistical significance and tendencies were considered at *p* < 0.05 and 0.05 ≤ *p* < 0.10, respectively. | | | | | | | | | |

**Supplemental Table S2.** Effects of organic acids on average daily feed intake and feed efficiency of nursery pigs (Trial#1)

|  | Treatment | | | |  | *p* value | | |
| --- | --- | --- | --- | --- | --- | --- | --- | --- |
|  | BA | SBA0.035 | SBA0.070 | SBA0.105 | SEM | Treatment | Linear | Quadratic |
| **ADFI, kg/d** |  |  |  |  |  |  |  |  |
| Phase1 | 0.08 | 0.09 | 0.09 | 0.08 | 0.01 | 0.49 | 0.73 | 0.37 |
| Phase2 | 0.40 | 0.42 | 0.45 | 0.39 | 0.02 | 0.19 | 0.98 | 0.11 |
| Phase3 | 0.87 | 0.84 | 0.89 | 0.81 | 0.03 | 0.22 | 0.22 | 0.19 |
| Overall | 0.54 | 0.53 | 0.57 | 0.48 | 0.02 | 0.01 | 0.43 | 0.10 |
| **G:F** |  |  |  |  |  |  |  |  |
| Phase1 | -0.04 | 0.07 | 0.17 | -0.13 | 0.15 | 0.45 | 0.95 | 0.51 |
| Phase2 | 0.62 | 0.75 | 0.68 | 0.65 | 0.04 | 0.12 | 0.78 | 0.17 |
| Phase3 | 0.66 | 0.69 | 0.65 | 0.68 | 0.02 | 0.42 | 0.22 | 0.29 |
| Overall | 0.61 | 0.68 | 0.65 | 0.66 | 0.03 | 0.03 | 0.09 | 0.03 |
| ADFI: average daily feed intake; G:F: feed efficiency; BA: basal diet+0.5% benzoic acid; SBA0.035: BA+0.035% butyrate; SBA0.070: BA+0.070% butyrate; SBA0.105: BA+0.105% butyrate. Phase1: d0 (weaning)-7; Phase2: d7-21; Phase3: d21-35; Overall: d0-35. Statistical significance and tendencies were considered at *p* < 0.05 and 0.05 ≤ *p* < 0.10, respectively. | | | | | | | | |

**Supplemental Table S3.** Effects of organic acids on average daily gain and body weight of nursery pigs (Trial#2)

|  | Treatment | | | | |  | *p* value | | | |
| --- | --- | --- | --- | --- | --- | --- | --- | --- | --- | --- |
|  | BA | SBA0.035 | SBA0.070 | SBA0.105 | NC | SEM | Treatment | Linear | Quadratic | BA vs NC |
| **ADG, kg** |  |  |  |  |  |  |  |  |  |  |
| Phase1 | -0.07 | -0.03 | -0.04 | -0.04 | -0.03 | 0.01 | 0.05 | 0.04 | 0.07 | 0.01 |
| Phase2 | 0.23 | 0.26 | 0.24 | 0.27 | 0.25 | 0.01 | 0.19 | 0.08 | 0.77 | 0.27 |
| Phase3 | 0.52 | 0.53 | 0.53 | 0.51 | 0.49 | 0.02 | 0.35 | 0.45 | 0.35 | 0.16 |
| Overall | 0.32 | 0.34 | 0.33 | 0.33 | 0.32 | 0.01 | 0.48 | 0.59 | 0.27 | 0.91 |
| **BW, kg** |  |  |  |  |  |  |  |  |  |  |
| Baseline | 4.71 | 4.60 | 4.71 | 4.68 | 4.74 | 0.21 | 0.39 | 0.95 | 0.40 | 0.75 |
| Phase1 | 4.25 | 4.42 | 4.45 | 4.43 | 4.51 | 0.20 | 0.03 | 0.03 | 0.09 | <0.01 |
| Phase2 | 7.47 | 8.11 | 7.84 | 8.21 | 7.99 | 0.31 | 0.07 | 0.02 | 0.48 | 0.06 |
| Phase3 | 17.44 | 18.21 | 17.91 | 17.86 | 17.40 | 0.55 | 0.54 | 0.57 | 0.29 | 0.95 |

ADG: average daily gain; BW: body weight; NC: basal diet; BA: basal diet+0.5% benzoic acid; SBA0.035: BA+0.035% butyrate; SBA0.070: BA+0.070% butyrate; SBA0.105: BA+0.105% butyrate. Phase1: d0 (weaning)-7; Phase2: d7-21; Phase3: d21-40; Overall: d0-40. Statistical significance and tendencies were considered at *p* < 0.05 and 0.05 ≤ *p* < 0.10, respectively.

**Supplemental Table S4.** Effects of organic acids on average daily feed intake and feed efficiency of nursery pigs (Trial#2)

|  | Treatment | | | | |  | *p* value | | | |  |
| --- | --- | --- | --- | --- | --- | --- | --- | --- | --- | --- | --- |
|  | BA | SBA0.035 | SBA0.070 | SBA0.105 | NC | SEM | Treatment | Linear | Quadratic | BA vs NC | |
| **ADFI, kg** | | |  |  |  |  |  |  |  |  |  |
| Phase1 | 0.06 | 0.10 | 0.08 | 0.09 | 0.09 | 0.00 | 0.03 | 0.20 | 0.05 | 0.01 |  |
| Phase2 | 0.28 | 0.33 | 0.30 | 0.31 | 0.31 | 0.02 | 0.09 | 0.21 | 0.14 | 0.06 |  |
| Phase3 | 0.75 | 0.84 | 0.79 | 0.79 | 0.75 | 0.03 | 0.05 | 0.39 | 0.08 | 0.89 |  |
| Overall | 0.46 | 0.54 | 0.49 | 0.50 | 0.48 | 0.02 | 0.03 | 0.25 | 0.05 | 0.34 |  |
| **G:F** |  |  |  |  |  |  |  |  |  |  |  |
| Phase1 | -1.38 | -0.33 | -0.55 | -0.48 | -0.35 | 0.24 | 0.02 | 0.03 | 0.05 | 0.00 |  |
| Phase2 | 0.79 | 0.76 | 0.79 | 0.83 | 0.78 | 0.03 | 0.45 | 0.19 | 0.23 | 0.75 |  |
| Phase3 | 0.70 | 0.63 | 0.68 | 0.64 | 0.66 | 0.02 | 0.05 | 0.12 | 0.23 | 0.12 |  |
| Overall | 0.68 | 0.62 | 0.67 | 0.65 | 0.65 | 0.02 | 0.26 | 0.65 | 0.28 | 0.33 |  |

ADFI: average daily feed intake; G:F: feed efficiency; NC: basal diet; BA: basal diet+0.5% benzoic acid; SBA0.035: BA+0.035% butyrate; SBA0.070: BA+0.070% butyrate; SBA0.105: BA+0.105% butyrate. Phase1: d0 (weaning)-7; Phase2: d7-21; Phase3: d21-40; Overall: d0-40. Statistical significance and tendencies were considered at *p* < 0.05 and 0.05 ≤ *p* < 0.10, respectively.

**Supplemental Table S5.** Effects of organic acids on complete blood count of nursery pigs (Trial#2)

|  | | Treatment | | | | | |  | *p* value | | | | |  |
| --- | --- | --- | --- | --- | --- | --- | --- | --- | --- | --- | --- | --- | --- | --- |
|  | | BA | SBA0.035 | SBA0.070 | | SBA0.105 | NC | SEM | Treatment | Treatment*Day | Linear | Quadratic | BA vs NC |  |
| **Concentration, k/µl** | |  |  |  |  | |  |  |  |  |  |  |  |  |
| WBC | | 14.21 | 15.24 | 14.57 | 13.11 | | 14.56 | 0.70 | 0.27 | 0.47 | 0.19 | 0.07 | 0.71 |  |
| Neutrophil | | 6.59 | 7.31 | 6.90 | 6.15 | | 7.09 | 0.47 | 0.43 | 0.55 | 0.40 | 0.11 | 0.44 |  |
| Lymphocyte | | 5.99 | 6.28 | 5.76 | 5.55 | | 5.95 | 0.33 | 0.29 | 0.24 | 0.09 | 0.31 | 0.90 |  |
| Monocyte | | 0.48 | 0.44 | 0.43 | 0.41 | | 0.38 | 0.04 | 0.47 | 0.39 | 0.22 | 0.85 | 0.07 |  |
| Eosinophil | | 1.09 | 1.13 | 1.40 | 0.91 | | 1.08 | 0.15 | 0.26 | 0.39 | 0.68 | 0.08 | 0.94 |  |
| Basophil | | 0.05 | 0.06 | 0.07 | 0.06 | | 0.05 | 0.01 | 0.74 | 0.71 | 0.34 | 0.53 | 0.97 |  |
| **Percentage over WBC** | |  |  |  |  | |  |  |  |  |  |  |  |  |
| Neutrophil | | 44.15 | 44.17 | 43.94 | 44.36 | | 44.75 | 1.70 | 0.99 | 0.97 | 0.95 | 0.88 | 0.74 |  |
| Lymphocyte | | 44.98 | 45.87 | 44.12 | 45.82 | | 45.59 | 1.91 | 0.95 | 0.86 | 0.92 | 0.82 | 0.81 |  |
| Monocyte | | 3.32 | 2.93 | 2.95 | 3.20 | | 2.71 | 0.25 | 0.46 | 0.39 | 0.76 | 0.21 | 0.09 |  |
| Eosinophil | | 7.22 | 6.70 | 8.78 | 6.36 | | 6.73 | 0.68 | 0.10 | 0.28 | 0.87 | 0.16 | 0.61 |  |
| Basophil | | 0.35 | 0.34 | 0.37 | 0.39 | | 0.31 | 0.04 | 0.74 | 0.83 | 0.44 | 0.72 | 0.51 |  |
| NLR | | 1.10 | 1.18 | 1.20 | 1.10 | | 1.17 | 0.10 | 0.87 | 0.86 | 0.93 | 0.29 | 0.53 |  |
| RBC, M/µl | | 6.64 | 6.91 | 6.82 | 6.88 | | 7.14 | 0.24 | 0.66 | 0.68 | 0.56 | 0.65 | 0.13 |  |
| Hemoglobin, g/dL | | 6.94 | 7.13 | 6.75 | 6.92 | | 6.88 | 0.29 | 0.89 | 0.67 | 0.71 | 0.96 | 0.87 |  |
| Hematocrit, % | | 28.19 | 29.22 | 28.68 | 28.57 | | 28.96 | 1.08 | 0.95 | 0.48 | 0.89 | 0.55 | 0.56 |  |
| MCV | | 42.34 | 42.03 | 41.72 | 41.11 | | 40.32 | 0.56 | 0.09 | 0.03 | 0.12 | 0.78 | 0.01 |  |
| MCH, Pg | | 10.89 | 10.13 | 9.65 | 9.74 | | 9.43 | 0.36 | 0.04 | 0.03 | 0.02 | 0.24 | 0.01 |  |
| MCHC, g/dL | | 25.07 | 23.83 | 22.88 | 23.22 | | 23.14 | 0.65 | 0.12 | 0.25 | 0.03 | 0.22 | 0.04 |  |
| RDW, % | | 28.71 | 28.41 | 27.89 | 28.18 | | 28.12 | 0.40 | 0.65 | 0.18 | 0.24 | 0.47 | 0.29 |  |
| PLT, k/µl | | 414.87 | 358.92 | 365.32 | 324.12 | | 382.69 | 29.90 | 0.14 | 0.68 | 0.02 | 0.76 | 0.36 |  |
| MPV, fL | | 8.28 | 7.58 | 7.92 | 8.01 | | 7.87 | 0.38 | 0.78 | 0.63 | 0.78 | 0.30 | 0.44 |  |
|  | NC: basal diet; BA: basal diet+0.5% benzoic acid; SBA0.035: BA+0.035% butyrate; SBA0.070: BA+0.070% butyrate; SBA0.105: BA+0.105% butyrate. Statistically significant value was *p* ≤ 0.05 and values tended to be significant at 0.05 < *p* ≤ 0.10.  Mean corpuscular volume (MCV): average volume of red blood cell | | | | | | | | | | | | | |
|  | Mean corpuscular hemoglobin (MCH): average mass of hemoglobin per red blood cell | | | | | | | | | | | | | |
|  | Mean corpuscular hemoglobin concentration (MCHC): a measure of the average concentration of hemoglobin inside a single red blood cell | | | | | | | | | | | | | |
|  | Red cell distribution width (RDW): measurement of the range in the volume and size of red blood cell | | | | | | | | | | | | | |
|  | PLT: platelet | | | | | | | | | | | | | |
|  | Mean platelet volume (MPV): average size of platelets | | | | | | | | | | | | | |

**Supplemental Table S6.** Effects of organic acids on volatile fatty acid concentration in fecal samples (Trial#2)

|  | Treatment | | | | | | | *p* value | | | | | | | | |  |
| --- | --- | --- | --- | --- | --- | --- | --- | --- | --- | --- | --- | --- | --- | --- | --- | --- | --- |
|  | BA | SBA0.035 | SBA0.070 | SBA0.105 | | NC | | SEM | Treatment | | Linear | | Quadratic | | BA vs NC | | |
| **Absolute conc., mM** |  |  | | |  | |  |  | |  | |  |  |  | |  |  |
| Acetate | 36.70 | 37.14 | 39.36 | 37.07 | | 39.53 | | 1.34 | 0.40 | | 0.97 | | 0.18 | | 0.14 | |  |
| Propionate | 17.43 | 17.67 | 19.35 | 17.53 | | 17.24 | | 0.90 | 0.48 | | 0.91 | | 0.12 | | 0.88 | |  |
| Butyrate | 12.57 | 13.05 | 14.87 | 12.60 | | 12.85 | | 0.89 | 0.34 | | 0.72 | | 0.07 | | 0.83 | |  |
| Iso-butyrate | 1.82 | 1.71 | 1.87 | 1.58 | | 1.71 | | 0.13 | 0.60 | | 0.50 | | 0.19 | | 0.55 | |  |
| Valerate | 3.97 | 3.91 | 4.33 | 3.84 | | 3.99 | | 0.31 | 0.81 | | 0.86 | | 0.23 | | 0.97 | |  |
| Iso-valerate | 2.72 | 2.49 | 2.66 | 2.24 | | 2.45 | | 0.23 | 0.60 | | 0.44 | | 0.29 | | 0.42 | |  |
| Total VFA | 75.21 | 75.97 | 82.44 | 74.85 | | 77.76 | | 3.20 | 0.45 | | 0.81 | | 0.08 | | 0.58 | |  |
| **Percentage of total VFA** |  |  |  |  | |  | |  |  | |  | |  | |  | |  |
| Acetate | 49.06 | 49.22 | 47.71 | 49.88 | | 50.95 | | 1.12 | 0.36 | | 0.68 | | 0.19 | | 0.24 | |  |
| Propionate | 23.16 | 23.20 | 23.46 | 23.36 | | 22.16 | | 0.49 | 0.34 | | 0.81 | | 0.76 | | 0.15 | |  |
| Butyrate | 16.53 | 16.99 | 18.08 | 16.71 | | 16.42 | | 0.69 | 0.45 | | 0.78 | | 0.15 | | 0.91 | |  |
| Iso-butyrate | 2.42 | 2.24 | 2.26 | 2.10 | | 2.19 | | 0.13 | 0.53 | | 0.46 | | 0.56 | | 0.23 | |  |
| Valerate | 5.22 | 5.09 | 5.25 | 5.00 | | 5.13 | | 0.24 | 0.94 | | 0.77 | | 0.47 | | 0.78 | |  |
| Iso-valerate | 3.61 | 3.26 | 3.23 | 2.95 | | 3.15 | | 0.25 | 0.46 | | 0.40 | | 0.69 | | 0.20 | |  |

NC: basal diet; BA: basal diet+0.5% benzoic acid; SBA0.035: BA+0.035% butyrate; SBA0.070: BA+0.070% butyrate; SBA0.105: BA+0.105% butyrate. Statistical significance and tendencies were considered at *p* < 0.05 and 0.05 ≤ *p* < 0.10, respectively.
